# High Salt Cross-Protects *Escherichia coli* from Antibiotic Treatment through Increasing Efflux Pump Expression

**DOI:** 10.1128/mSphere.00095-18

**Published:** 2018-04-11

**Authors:** Manlu Zhu, Xiongfeng Dai

**Affiliations:** a School of Life Sciences, Central China Normal University, Wuhan, China; JMI Laboratories

**Keywords:** antibiotic susceptibility, cross-protection, efflux pumps, high salt

## Abstract

Environmental stresses often co-occur when bacteria encounter antibiotic treatment inside the human body. The cellular response to environmental stressors can alter the global gene expression pattern of bacteria. However, the relationship between the cellular stress response and antibiotic susceptibility remains poorly understood. Here we studied the effect of high salt, an important environmental stress condition inside the human body, on bacterial susceptibility to antibiotics. We found that high salt reduces the susceptibility of Escherichia coli to tetracycline and chloramphenicol, leading to a cross-protection effect. The cross-protection effect originates from the increased AcrAB-TolC efflux pump expression level under high-salt conditions. Our study demonstrates that stress-induced gene expression alterations can cross-protect bacteria from antibiotic treatment and should thus be considered when investigating antibiotic susceptibility and applying antimicrobial treatment.

**IMPORTANCE** Environmental stresses often co-occur when bacteria confront antibiotic treatment. We provide a clear example that a natural stress condition (high salt) can cross-protect bacteria from antibiotic treatment by triggering the bacterial stress response program (elevated AcrAB-TolC efflux pump expression). Our study highlights the importance of taking the co-occurrence of bacterial environmental stresses into consideration when investigating antibiotic susceptibility and applying antimicrobial treatment.

## Abstract (precis)

Environmental stresses often co-occur when bacteria confront antibiotic treatment. We provide a clear example that a natural stress condition (high salt) can cross-protect bacteria from antibiotic treatment by triggering the bacterial stress response program (elevated AcrAB-TolC efflux pump expression). Our study highlights the importance of taking the co-occurrence of bacterial environmental stresses into consideration when investigating antibiotic susceptibility and applying antimicrobial treatment.

## INTRODUCTION

In nature, bacteria need to cope with many harsh environmental conditions, such as nutrient depletion, high temperature, oxidative stress, acidity, and a high salt concentration (1). Environmental stresses usually cause either a reduced growth rate or reduced viability. Under these circumstances, bacteria must initiate a stress response program to enable the survival of the population (1–4). Cellular stress response can alter the global pattern of bacterial gene expression, which is usually associated with changes in the levels of hundreds of proteins (5–8). For example, the regulon of the general stress response sigma factor RpoS contains >100 genes, which are upregulated under stress conditions (9–11).

Environmental stresses often co-occur when bacteria confront antibiotic treatment inside the human body (12). However, it remains largely unclear how the stress response program can affect the susceptibility of bacteria to antibiotic treatment. A high salt concentration is a common environmental stress condition for bacteria. The high salt contained in a “Western diet” is closely related to numerous human disorders, including hypertension, cardiovascular disease, autoimmune disease, and cognitive dysfunction (13–16). A recent study has demonstrated that the effect of a high salt concentration on the gut microbiome is closely implicated in salt-mediated human disorders (15), indicating that a high salt concentration can significantly affect the basic physiology of bacteria inside the human body. A high salt concentration imposes hyperosmotic stress on bacteria. When confronting a high salt concentration, bacteria must initiate a stress response to tackle the severe loss of water and turgor pressure (2, 17–20). Cellular stress response enables Escherichia coli to accumulate osmolytes such as potassium, glutamate, and trehalose to maintain the balance of external and internal osmolarity (21). This process helps E. coli regain water and turgor pressure to support growth.

Though a high salt concentration is proposed to significantly affect microbial physiology, it remains unclear whether a high salt concentration can affect the antibiotic susceptibility of bacteria. Here, we find that a high salt concentration cross-protects E. coli from antibiotic treatment, which results from increased AcrAB-TolC efflux pump expression under high-salt conditions.

## RESULTS AND DISCUSSION

### Decreased antibiotic susceptibility of E. coli under high-salt conditions.

**FIG 1:**
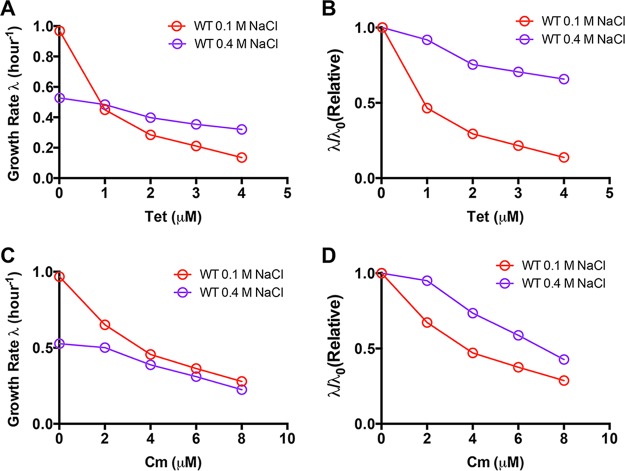
A high salt concentration cross-protects E. coli from antibiotic treatment. (A) Growth rate of E. coli upon tetracycline (Tet) treatment in glucose medium containing 0.1 or 0.4 M NaCl. (B) Relative change in the growth rate upon tetracycline treatment. λ_0_ denotes the growth rate of E. coli in drug-free medium. (C) Growth rate of E. coli upon chloramphenicol (Cm) treatment in glucose medium containing 0.1 or 0.4 M NaCl. (D) Relative change in the growth rate upon chloramphenicol treatment. λ_0_ denotes the growth rate of E. coli in drug-free medium. WT, wild type.

To investigate the effect of high-salt conditions on antibiotic susceptibility, we measured the antibiotic growth inhibition curve of E. coli cells under both normal conditions (0.1 M NaCl in glucose minimal medium) and high-salt conditions (0.4 M NaCl in glucose minimal medium). Two well-known ribosome-targeting antibiotics, tetracycline (binds to the 30S ribosomal subunit) and chloramphenicol (binds to the 50S ribosomal subunit), were used to study drug susceptibility (22). The exponential growth rate of E. coli cells was plotted as a function of the antibiotic concentration in the medium. As shown in Fig. 1A, the bacterial growth rate dramatically drops with increasing concentrations of tetracycline under normal conditions (red symbols). Under high-salt conditions, though the growth rate is much lower than that under normal conditions in drug-free medium (0.52/h at 0.4 M NaCl versus 0.96/h at 0.1 M NaCl), it drops very slowly with increasing concentrations of tetracycline (purple symbols). The growth rate under high-salt conditions exceeds that under normal conditions when the tetracycline concentration is >1 µM. This result demonstrates that high salt can lead to cross-protection against antibiotics. For a direct comparison, we made the inhibition curve of the relative growth rate (the growth rate is normalized by the growth rate in drug-free medium, λ/λ_0_) (Fig. 1B). The tetracycline susceptibility of E. coli under high-salt conditions is indeed much weaker than that under normal conditions. The bacterial growth rate drops by ~60% at 1 µM tetracycline under normal conditions, while under high-salt conditions, it drops by only ~35%, even at 4 µM tetracycline. Similarly, high salt also reduces the chloramphenicol susceptibility of E. coli (Fig. 1C and D). The cross-protection effect against chloramphenicol is especially remarkable in glucose-6-phosphate minimal medium, where 4 µM chloramphenicol has no effect on the bacterial growth rate under high-salt conditions (Fig. S1).

10.1128/mSphere.00095-18.1 FIG S1 Chloramphenicol susceptibility in gluose-6-phosphate medium. (A) Growth rate of E. coli upon chloramphenicol (Cm) treatment in glucose-6-phosphate medium containing 0.1 or 0.4 M NaCl. (B) Relative change in the growth rate upon chloramphenicol treatment. λ_0_ refers to the growth rate of E. coli in drug-free medium. Download FIG S1, PDF file, 0.1 MB. Copyright © 2018 Zhu and Dai. 2018 Zhu and Dai This content is distributed under the terms of the Creative Commons Attribution 4.0 International license.

### Significant alterations in efflux pump and porin protein expression levels under high-salt conditions.

**FIG 2:**
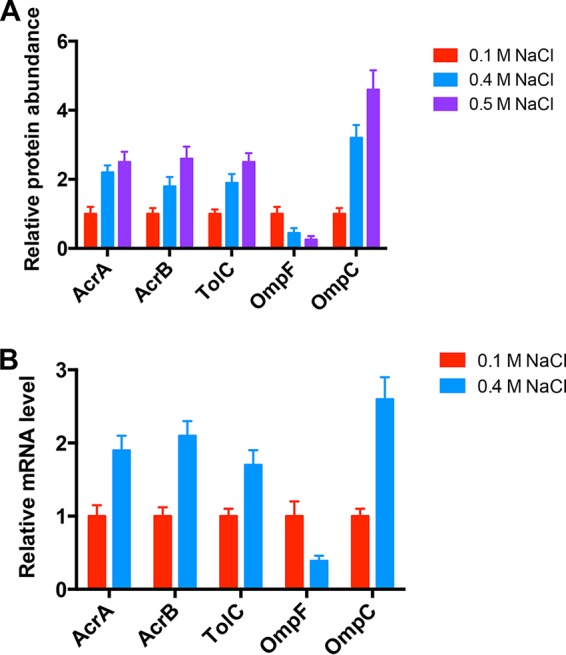
Efflux pump and porin gene expression levels under high-salt conditions. (A) Protein abundance determined by mass spectrometry. (B) mRNA levels determined by qPCR. Data obtained under normal conditions (0.1 M NaCl) were set as 1.

To investigate the origin of cross-protection against antibiotics mediated by high salt, we postulated that high salt could change the level of some specific proteins, which ultimately reduced the intracellular drug concentration. In principle, intracellular drug accumulation can be affected by drug permeation and drug efflux (23). Porin proteins OmpF and OmpC are among the most abundant outer membrane proteins and are proposed to be responsible for controlling drug influx into cells. On the other hand, AcrAB-TolC is the major multidrug efflux pump system that pumps a drug out of the cell to reduce its effective intracellular concentration (23–25). We applied quantitative mass spectrometry and real-time quantitative PCR (qPCR) to measure relative *ompF*, *ompC*, *acrA*, *acrB*, and *tolC* expression in normal and high-salt media. As shown in Fig. 2, the relative *acrA*, *acrB*, and *tolC* expression levels all strongly increase under high-salt conditions. The expression levels of two porin proteins exhibit the opposite trend under high-salt conditions. *ompF* expression strongly decreases while *ompC* expression increases remarkably under high-salt conditions. Overall, on the basis of the above-described results, the reduced antibiotic susceptibility that occurs under high-salt conditions may be attributed to increased drug efflux, decreased drug influx (mediated by OmpF), or both.

### Disappearance of cross-protection effect in efflux pump mutants.

**FIG 3:**
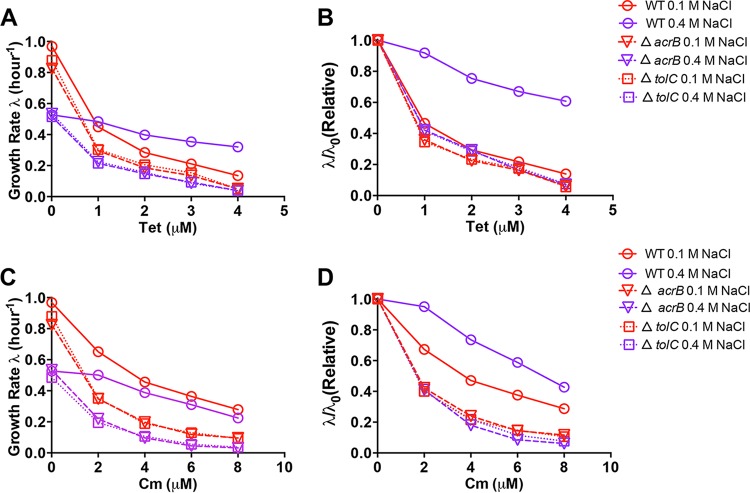
Antibiotic susceptibility of E. coli efflux pump mutants under high-salt conditions. (A) Growth rates of E. coli wild-type (WT), *acrB*-deficient, and *tolC*-deficient strains upon tetracycline (Tet) treatment in glucose medium containing 0.1 or 0.4 M NaCl. (B) Relative change in the growth rate upon tetracycline treatment. λ_0_ refers to the growth rate of E. coli in drug-free medium. (C) Growth rates of E. coli wild-type, *acrB*-deficient, and *tolC*-deficient strains upon chloramphenicol (Cm) treatment in glucose medium containing 0.1 or 0.4 M NaCl. (D) Relative change in the growth rate upon chloramphenicol treatment. λ_0_ refers to the growth rate of E. coli in drug-free medium.

**FIG 4:**
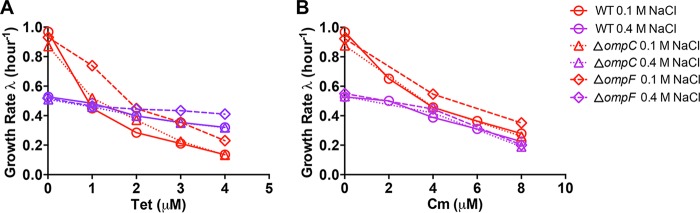
Antibiotic susceptibility of E. coli porin mutants under high-salt conditions. (A) Growth rates of E. coli wild-type (WT), *ompC*-deficient, and *ompF*-deficient strains upon tetracycline (Tet) treatment in glucose medium containing 0.1 or 0.4 M NaCl. (B) Growth rates of E. coli wild type, *ompC*-deficient, and *ompF*-deficient strains upon chloramphenicol (Cm) treatment in glucose medium containing 0.1 or 0.4 M NaCl.

We next characterized the antibiotic susceptibility of E. coli mutants deficient in either efflux pump or porin proteins (Fig. 3 and 4). Both the *acrB* and *tolC* mutants exhibit remarkably higher susceptibility to tetracycline and chloramphenicol than wild-type cells under both normal and high-salt conditions, confirming the important role of the AcrAB-TolC efflux pump in bacterial drug resistance. Strikingly, unlike the case of wild-type cells, the growth rates of *acrB* and *tolC* mutant cells under high-salt conditions also dramatically drop with increasing concentrations of tetracycline and chloramphenicol (Fig. 3A and C). The disappearance of the cross-protection effect is explicitly demonstrated in the relative growth rate inhibition curve; the relative growth rate inhibition curves of the *acrB* and *tolC* mutants under both normal and high-salt conditions almost completely overlap (Fig. 3B and D). This result indicates that the AcrAB-TolC efflux pump is related to the cross-protection against antibiotics mediated by high-salt conditions.

The growth inhibition curve of the *ompC* mutant was almost the same as that of wild-type cells (Fig. 4), indicating that the change in *ompC* expression is not related to the cross-protection effect under high-salt conditions. The *ompF* mutant is slightly less susceptible to tetracycline treatment than wild-type cells under both normal and high-salt conditions (Fig. 4A). However, the growth rate of the *ompF* mutant still exhibits a strong dependence on the tetracycline concentration under normal conditions (Fig. 4A). Moreover, the chloramphenicol susceptibility of the *ompF* mutant is similar to that of the *ompC* mutant and the wild-type strain under both normal and high-salt conditions (Fig. 4B). This indicates that the reduction of *ompF* expression under high-salt conditions contributes only marginally to cross-protection against antibiotics.

### Decreased antibiotic susceptibility of efflux pump overexpression strain.

**FIG 5:**
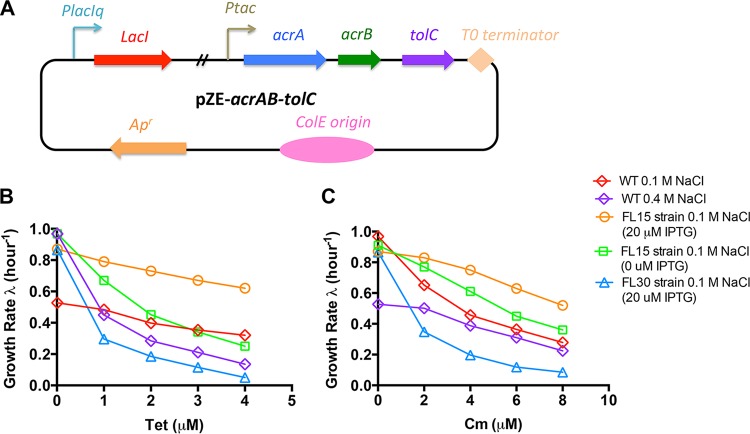
Decreased antibiotic susceptibility of E. coli efflux pump overexpression strain FL15. (A) pZE-*acrAB-tolC* vector for AcrAB-TolC efflux pump overexpression. The *tolC* gene, together with its RBS, is placed downstream of the *acrAB* operon so that the three genes form a single cistron. AcrAB-TolC efflux pump expression is driven by the P_*tac*_ promoter. The vector contains the p*lacII*^q^*-lacI* cassette for regulation of P_*tac*_ promoter expression. (B) Growth rate of E. coli efflux pump overexpression strain FL15 (*acrB*-deficient strain harboring pZE-*acrAB-tolC*) and control strain FL30 (*acrB*-deficient strain harboring pZE-*gfp*) upon tetracycline treatment under normal conditions (glucose medium containing 0.1 M NaCl). The data obtained with the wild-type (WT) strain at both 0.1 and 0.4 M NaCl are plotted together for comparison. (C) Growth rates of E. coli FL15 and FL30 strains upon chloramphenicol treatment under normal conditions (glucose medium containing 0.1 M NaCl). The data obtained with the wild-type strain at both 0.1 and 0.4 M NaCl are plotted together for comparison.

To further confirm that the cross-protection effect originates from the increased AcrAB-TolC efflux pump expression level, we studied whether artificial AcrAB-TolC efflux pump overexpression could also lead to decreased antibiotic susceptibility under normal conditions. We constructed an artificial AcrAB-TolC efflux pump overexpression vector (Fig. 5A). The *acrAB* operon was placed downstream of the P_*tac*_ promoter. In addition, the *tolC* gene, together with its native ribosome-binding site (RBS), was directly inserted downstream of the *acrAB* operon to form a single *acrAB-tolC* cistron. Therefore, the expression of *acrAB-tolC* is driven by the inducible P_*tac*_ promoter, which is under the regulation of a p*lacI*^q^*-lacI* cassette. The AcrAB-TolC overexpression vector pZE-*acrAB-tolC* was transformed into *acrB* and *tolC* mutants to obtain the FL15 and FL16 strains, respectively. We then measured the antibiotic growth inhibition curves of the FL15 and FL16 strains under normal conditions.

As shown in Fig. 5 and S2, elevated AcrAB-TolC efflux pump expression (20 µM isopropyl-β-d-thiogalactopyranoside [IPTG]) indeed reduces the antibiotic susceptibility of E. coli under normal conditions, mimicking the pattern of the growth inhibition curve of wild-type cells under high-salt conditions. Even in the absence of an inducer, the leaky AcrAB-TolC efflux pump expression driven by the P_*tac*_ promoter has mildly reduced the antibiotic susceptibility compared with that of wild-type cells. On the contrary, the FL30 and FL31 strains, which overexpress green fluorescent protein, used as the control are still as highly susceptible to antibiotic treatment as the *acrB* and *tolC* mutants. Overall, the above results strongly support the notion that the antibiotic cross-protection effect of high-salt conditions results from increased AcrAB-TolC efflux pump expression.

10.1128/mSphere.00095-18.2 FIG S2 Decreased antibiotic susceptibility of E. coli efflux pump overexpression strain FL16. (A) Growth rate of E. coli efflux pump overexpression strain FL16 (*tolC*-deficient strain harboring pZE-*acrAB-tolC*) and control strain FL31 (*tolC*-deficient strain harboring pZE-*gfp*) upon tetracycline (Tet) treatment under normal conditions (glucose medium containing 0.1 M NaCl). The data obtained with the wild-type (WT) strain at both 0.1 and 0.4 M NaCl are plotted together for comparison. (B) Growth rates of E. coli FL16 and FL31 strains upon chloramphenicol (Cm) treatment under normal conditions (glucose medium containing 0.1 M NaCl). The data obtained with the wild-type strain at both 0.1 and 0.4 M NaCl are plotted together for comparison. Download FIG S2, PDF file, 0.04 MB. Copyright © 2018 Zhu and Dai. 2018 Zhu and Dai This content is distributed under the terms of the Creative Commons Attribution 4.0 International license.

The functional AcrAB-TolC multidrug efflux pump requires all three proteins (25); therefore, the increased antibiotic tolerance should, in principle, require increased expression of all three efflux pump proteins, as found under high-salt conditions (Fig. 2). In support of this assumption, both the FL17 (*acrB* deficient and overexpressing AcrA and AcrB) and FL18 (*tolC* deficient and overexpressing TolC) strains fail to obtain higher antibiotic tolerance than wild-type cells (Fig. S3 and S4). This result indicates that it is the increase in all three efflux pump proteins (functional AcrAB-TolC efflux pump) that is indispensable for the antibiotic cross-protection effect of high-salt conditions.

10.1128/mSphere.00095-18.3 FIG S3 Antibiotic susceptibility of E. coli strain FL17 overexpressing AcrA and AcrB. (A) pZE-*acrAB* vector for overexpression of AcrA and AcrB proteins. (B) Growth rate of strain FL17 (*acrB*-deficient strain harboring pZE-*acrAB*) upon tetracycline (Tet) treatment under normal conditions (glucose medium containing 0.1 M NaCl). The data obtained with the wild-type (WT) strain at both 0.1 and 0.4 M NaCl are plotted together for comparison. (C) Growth rate of strain FL17 upon chloramphenicol (Cm) treatment under normal conditions (glucose medium containing 0.1 M NaCl). The data obtained with the wild-type strain at both 0.1 and 0.4 M NaCl are plotted together for comparison. Download FIG S3, PDF file, 0.1 MB. Copyright © 2018 Zhu and Dai. 2018 Zhu and Dai This content is distributed under the terms of the Creative Commons Attribution 4.0 International license.

10.1128/mSphere.00095-18.4 FIG S4 Antibiotic susceptibility of E. coli strain FL18 overexpressing TolC. (A) pZE-*tolC* vector for overexpression of TolC. (B) Growth rate of strain FL18 (*tolC*-deficient strain harboring pZE-*tolC*) upon tetracycline (Tet) treatment under normal conditions (glucose medium containing 0.1 M NaCl). The data obtained with the wild-type (WT) strain at both 0.1 and 0.4 M NaCl are plotted together for comparison. (C) Growth rate of strain FL18 upon chloramphenicol (Cm) treatment under normal conditions (glucose medium containing 0.1 M NaCl). The data obtained with the wild-type (WT) strain at both 0.1 and 0.4 M NaCl are plotted together for comparison. Download FIG S4, PDF file, 0.1 MB. Copyright © 2018 Zhu and Dai. 2018 Zhu and Dai This content is distributed under the terms of the Creative Commons Attribution 4.0 International license.

### Conclusion.

Our study demonstrates that a high salt concentration, a common environmental stress condition encountered by bacteria inside the human body, can cross-protect them from antibiotic treatment by triggering the stress response program. It was known before that nutrient limitation can also enhance bacterial antibiotic tolerance by increasing the fraction of persister cells in the bacterial population (26–29). The increased fraction of persister cells originates from the induction of toxin-antitoxin activity by ppGpp signaling under nutrient-limited conditions. The persister cells represent a subpopulation that can survive antibiotic treatment while they remain largely metabolically dormant. Instead, here we find that high-salt conditions can reduce bacterial susceptibility to antibiotics at the whole-population instead of the subpopulation level. Our study highlights the importance of taking the co-occurrence of environmental stressors into consideration when investigating antibiotic susceptibility and applying antimicrobial treatment. The antibiotic cross-protection mediated by environmental stresses inside the human body may accelerate the spread of bacterial drug resistance, which will further complicate the current severe drug resistance situation and should be taken into consideration in future clinical studies.

## MATERIALS AND METHODS

### Strains.

The strains used in this study included the wild-type K-12 NCM3722 strain (22, 30) and its derivatives including *acrB*, *tolC*, *ompF*, and *ompC* mutants and AcrAB-TolC overexpression strains FL15 and FL16. To construct the efflux pump- and porin protein-deficient forms of strain NCM3722, the *acrB*::*kan*, *tolC*::*kan*, *ompF*::*kan*, and *ompC*::*kan* alleles in related mutant strains from the Keio collection (31) were transferred into NCM3722 through P1 transduction to generate the four mutant strains, respectively.

To construct the AcrAB-TolC overexpression vector pZE-*acrAB-tolC*, a p*lacII*^q^*-lacI* cassette was first inserted into the AatII/XhoI sites of the pZE11-luc vector (32); a P_*tac*_ promoter was PCR amplified and cloned into the XhoI/KpnI sites to replace the P_*LtetO-1*_ promoter of the native vector; the coding sequence of the *acrAB* operon was then inserted downstream of the P_*tac*_ promoter at the KpnI/XbaI sites, resulting in pZE-*acrAB*; and finally, the coding sequence of the *tolC* gene, together with its RBS, was inserted into the XbaI sites of pZE-*acrAB* to form a single cistron with the *acrAB* operon, generating the pZE-*acrAB-tolC* vector. The pZE-*acrAB-tolC* vector was then transformed into the *acrB* and *tolC* mutants to generate the FL15 and FL16 strains, respectively. The PCR reagents used in this study were Golden PCR mix (green) and T5 super PCR mix (Tsingke BioTech Co., China).

The pZE-*acrAB* vector was transformed into the *acrB*::*kan* mutant strain, resulting in the FL17 strain. The *acrAB* operon in the pZE-*acrAB* vector was also replaced with the *tolC* and *gfp* genes, resulting in the pZE-*tolC* and pZE-*gfp* vectors, respectively. The pZE-*tolC* vector was transformed into the *tolC*::*kan* mutant strain to obtain the FL18 strain. The pZE-*gfp* vector was transformed into the *acrB* and *tolC* mutants to generate the FL30 and FL31 strains, respectively.

### Growth medium.

The growth medium used in this study was either morpholinepropanesulfonic acid (MOPS)-buffered glucose minimal medium (used in most of our experiments) or glucose-6-phosphate minimal medium (used solely in the experiment described in Fig. S1) as described by Cayley et al. (17). The final NaCl concentrations in normal and high-salt media were 0.1 and 0.4 M, respectively. The growth media were supplemented with different concentrations of tetracycline and chloramphenicol (Solarbio Life Sciences, Beijing). The LB medium used to grow seed cultures contained 0.5% yeast extract, 1% tryptone, and 1% sodium chloride (33).

### Cell growth.

Cell growth experiments were always performed in a 37°C air bath shaker (220 rpm). Seed culture was grown in LB medium (Coolaber Biotech, Beijing) for several hours and inoculated into the minimal medium supplemented with antibiotics for overnight growth as precultures. On the next day, the precultures were inoculated into the same antibiotic-containing minimal medium at an initial optical density at 600 nm (OD_600_) of ~0.01 as the final experimental culture. During the cell growth procedures, six to eight OD_600_ data points in the range of 0.05 to 0.5 were measured by a GENESYS 30 visible spectrophotometer (Thermo Fisher Scientific) at different time points to generate an exponential-phase growth curve for calculation of the bacterial growth rate.

### Real-time qPCR.

Two milliliters of cell culture (OD_600_ of ~0.4) was used for total RNA extraction with the RNAprep Bacterial kit (Tiangen, China). The total RNA concentration was then measured by NanoDrop spectrophotometry. A 1-µg sample of total RNA was used for cDNA synthesis via reverse transcription with TranScript cDNA Synthesis SuperMix (Tiangen, China). The qPCRs were performed with a SuperReal Premix SYBR green Plus kit (Yeasen Biotech, Shanghai, China) in accordance with the manual. The qPCRs were carried out in a Bio-Rad CFX96 Touch real-time PCR system with the following protocol: 95°C for 15 min, followed by 40 cycles of 95°C for 30 s, 60°C for 30 s, and 72°C for 30 s. The *ftsZ* housekeeping gene was used as the internal reference.

### Measurement of protein abundance.

The abundances of porin and efflux pump proteins were measured by quantitative mass spectrometry as described by Hui et al. and Dai et al. (5, 20).
