# Erratum for Zhu and Dai, “High Salt Cross-Protects Escherichia coli from Antibiotic Treatment through Increasing Efflux Pump Expression”

**DOI:** 10.1128/mSphere.00267-18

**Published:** 2018-05-30

**Authors:** Manlu Zhu, Xiongfeng Dai

**Affiliations:** a School of Life Sciences, Central China Normal University, Wuhan, China

## ERRATUM

Volume 3, no. 2, e00095-18, 2018, https://doi.org/10.1128/mSphere.00095-18. On page 7 of the PDF, the first two paragraphs of the Acknowledgments section should read as follows.

We acknowledge discussions with the Terence Hwa lab at the University of California, San Diego, where this work was initiated.

This work was supported by the National Natural Science Foundation of China (grants 31700039 to M.Z. and 31700089 to X.D.), by startup funding from the Central China Normal University, and by U.S. National Institutes of Health grant R01GM095903 through Terence Hwa. M.Z. and X.D. especially thank the Institute of Science and Technology Development of the Central China Normal University for funding support.

