# Supplementary figures and images for: High Salt Cross-Protects Escherichia coli from Antibiotic Treatment through Increasing Efflux Pump Expression
Source: mSphere. 2018 Apr 11;3(2):e00095-18. doi: 10.1128/mSphere.00095-18 (PMC5909119; doi:10.1128/mSphere.00095-18)

**A****Glucose-6-phosphate**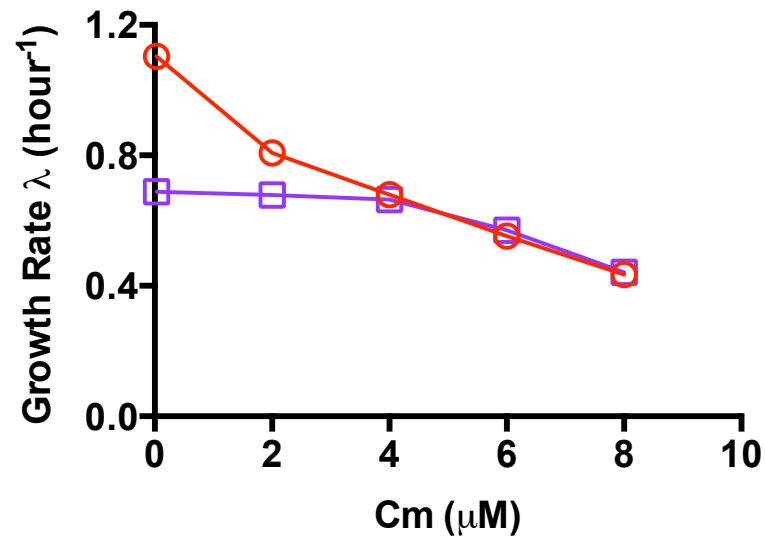**B****Glucose-6-phosphate**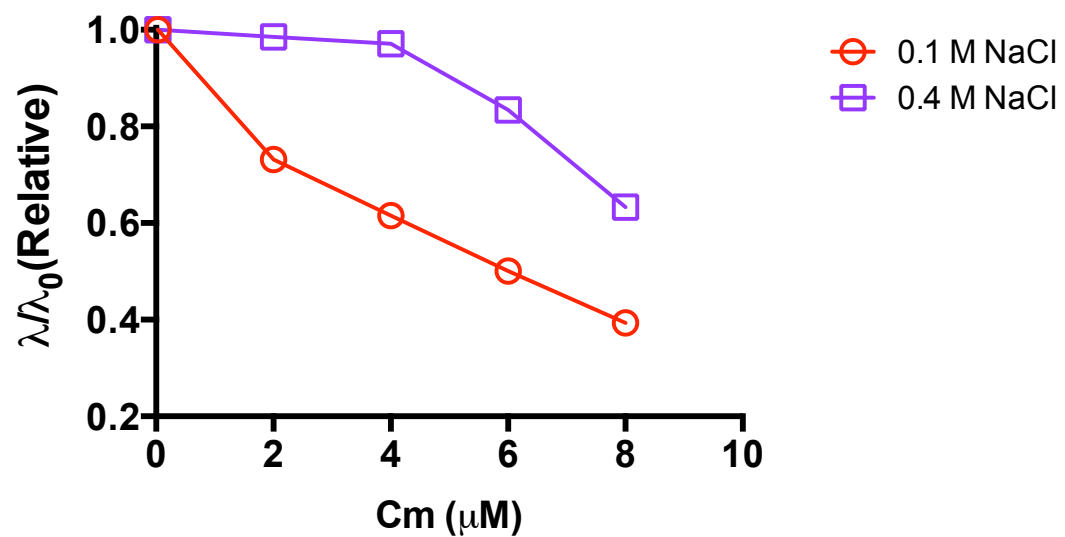

Supplement: FIG S1 [file sph002182510sf1.pdf]

**A**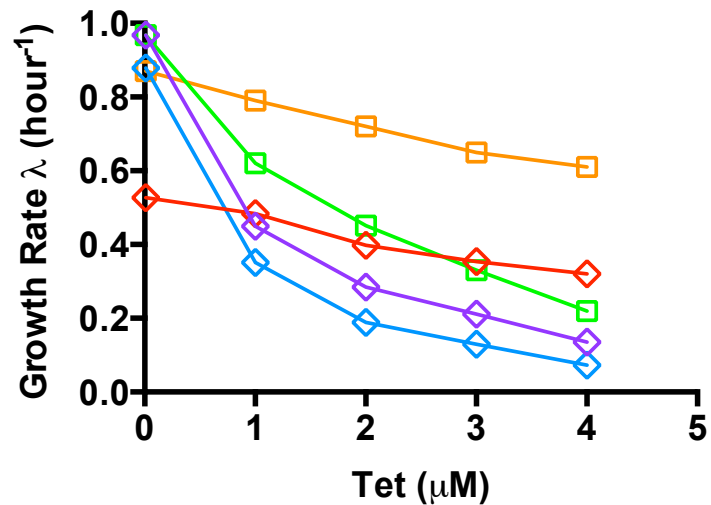**B**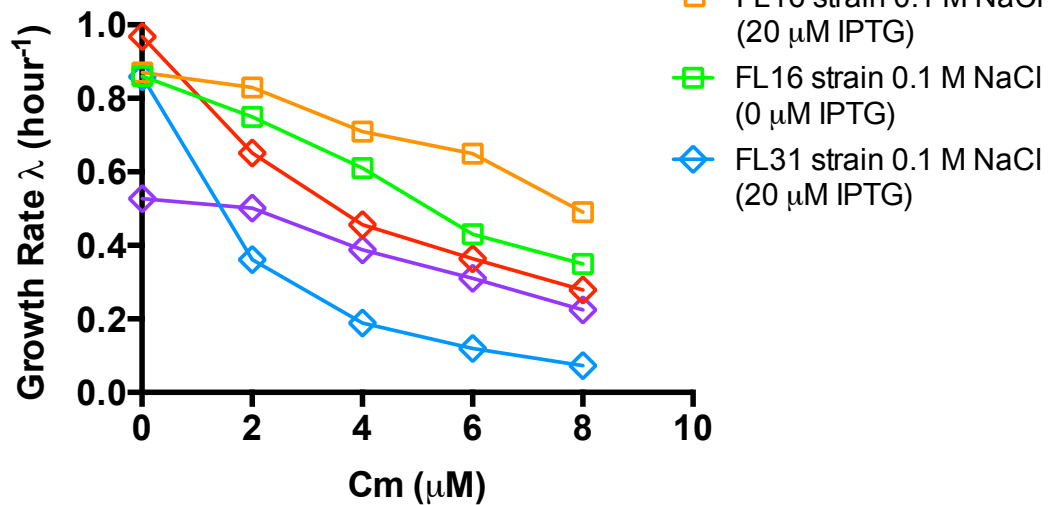

Supplement: FIG S2 [file sph002182510sf2.pdf]

**A**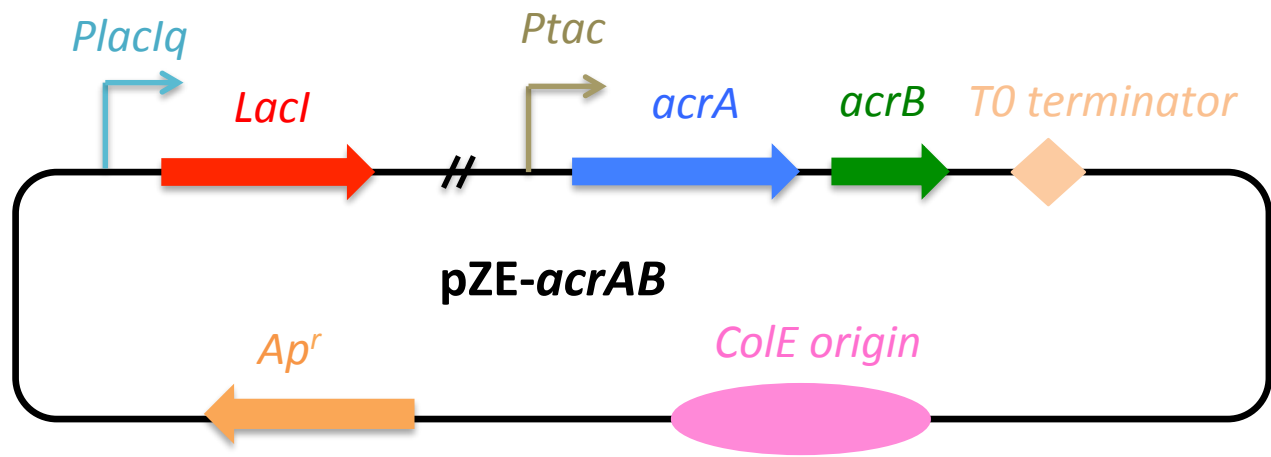**B**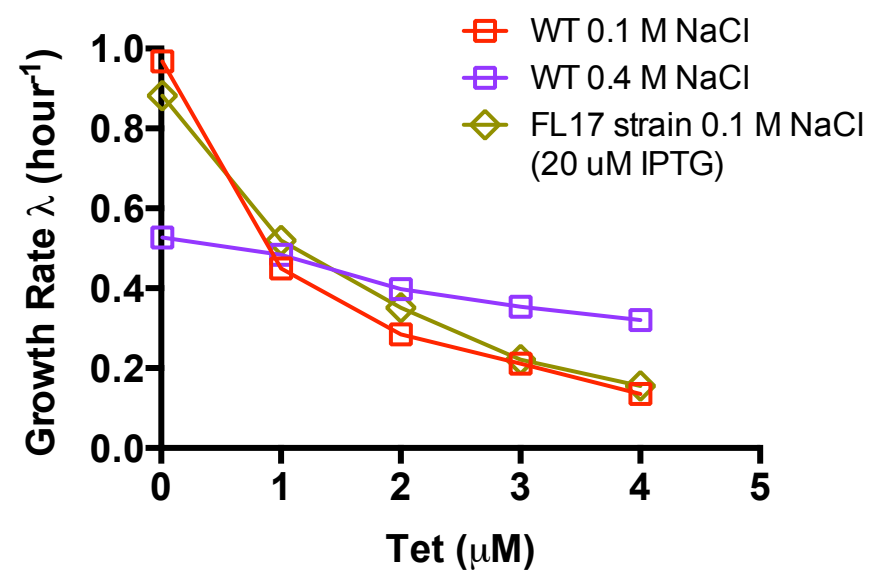**C**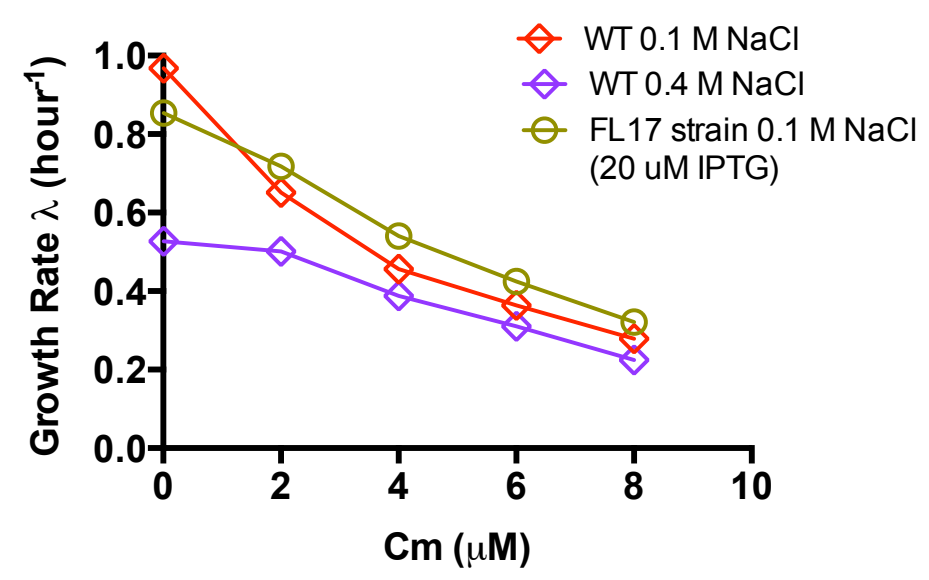

Supplement: FIG S3 [file sph002182510sf3.pdf]

**A**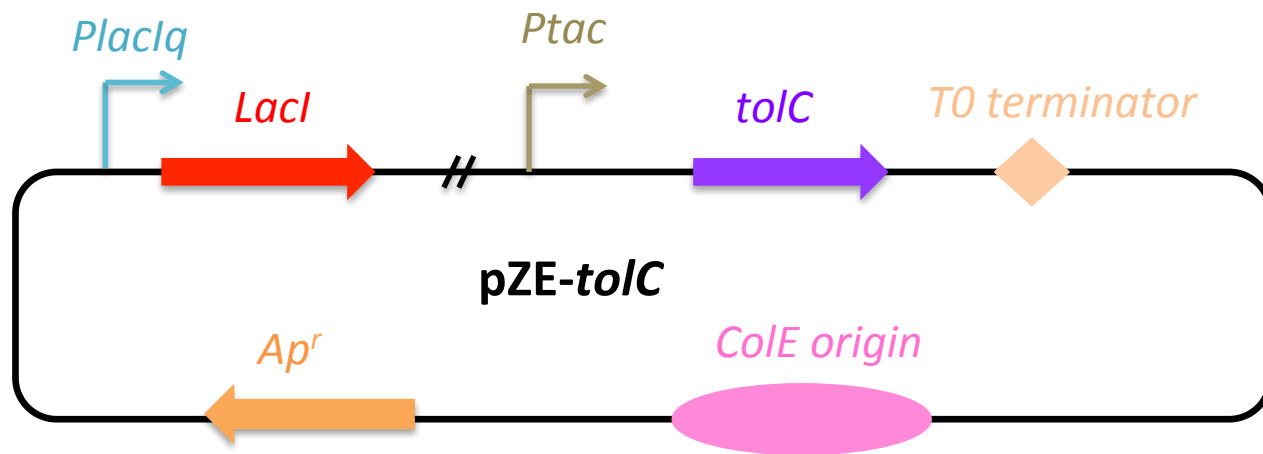**B**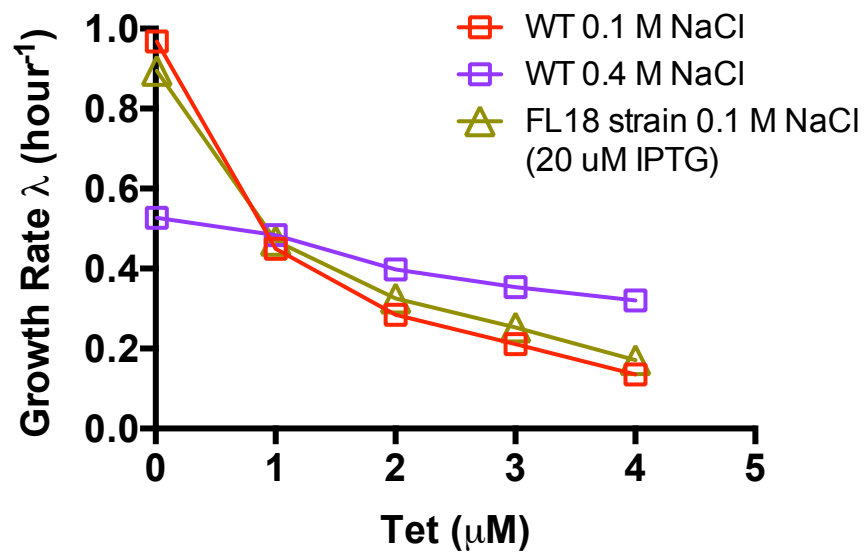**C**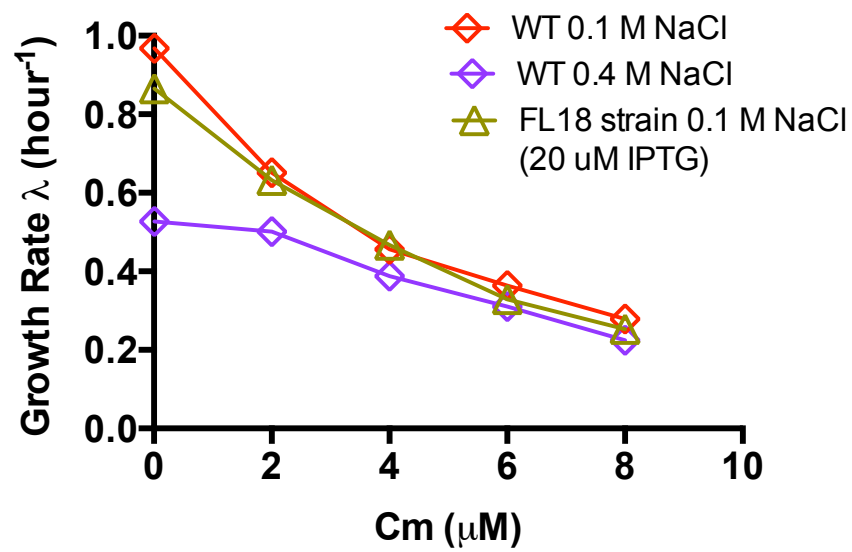

Supplement: FIG S4 [file sph002182510sf4.pdf]
